# Multi-Parametric Birefringence Control in Ultrashort-Pulse Laser-Inscribed Nanolattices in Fluorite

**DOI:** 10.3390/nano13061133

**Published:** 2023-03-22

**Authors:** Sergey Kudryashov, Alexey Rupasov, Mikhail Smayev, Pavel Danilov, Evgeny Kuzmin, Irina Mushkarina, Alexey Gorevoy, Anna Bogatskaya, Alexander Zolot’ko

**Affiliations:** 1 Lebedev Physical Institute, 119991 Moscow, Russia; 2 Physics Department, Moscow State University, 119991 Moscow, Russia

**Keywords:** fluorite, ultrashort-pulse laser, direct laser inscription, nanolattices, birefringence

## Abstract

An ultrashort-pulse laser inscription of embedded birefringent microelements was performed inside bulk fluorite in pre-filamentation (geometrical focusing) and filamentation regimes as a function of laser wavelength, pulsewidth and energy. The resulting elements composed of anisotropic nanolattices were characterized by retardance (*Ret*) and thickness (*T*) quantities, using polarimetric and 3D-scanning confocal photoluminescence microscopy, respectively. Both parameters exhibit a monotonous increase versus pulse energy, going over a maximum at 1-ps pulsewidth at 515 nm, but decrease versus laser pulsewidth at 1030 nm. The resulting refractive-index difference (RID) Δ*n* = *Ret*/*T* ~ 1 × 10^−3^ remains almost constant versus pulse energy and slightly decreases at a higher pulsewidth, generally being higher at 515 nm. The birefringent microelements were visualized using scanning electron microscopy and chemically characterized using energy-dispersion X-ray spectroscopy, indicating the increase of calcium and the contrary decrease of fluorine inside them due to the non-ablative inscription character. Dynamic far-field optical diffraction of the inscribing ultrashort laser pulses also demonstrated the accumulative inscription character, depending on the pulse energy and the laser exposure. Our findings revealed the underlying optical and material inscription processes and demonstrated the robust longitudinal homogeneity of the inscribed birefringent microstructures and the facile scalability of their thickness-dependent retardance.

## 1. Introduction

Micro-bits made of regular birefringent nanolattices (often also called nanogratings) and inscribed using ultrashort—femto- or pico-second (fs, ps)—laser pulses in bulk dielectric materials [1] emerged as key enabling building blocks of interferential-polarizing micro-optical devices [2,3], strongly competing with ultramodern metasurfaces in terms of intrinsic optical strength, mechanical durability and micro-integration capacity. Such nanolattices are known to be the product of ultrashort-pulse laser–dielectric interactions involving near-field scattering, plasmonics and material modification [4,5,6]. The resulting one-dimensional interferential backscattered [4] or plasmonic [5,6] “hot spots” imprinted in the dielectric materials as nanoscale periodic structural modification stripes [7,8,9,10], exhibit anisotropic periodical modulation of the refractive index (form birefringence) [1,3]. A buried dynamic near-critical electron-hole plasma, supporting interfacial plasmon-polaritons during the bulk refractive-index difference (RID) laser inscription, could be produced either in pre-filamentation (linear/geometrical focusing) [11] or in filamentation (non-linear self-focusing) regimes [12]. Importantly, nano/microscale damage morphologies—nano- [1,3] and microcavities [13,14], microtracks [12], etc., accompanying the pre- or filamentation regimes, could be managed by laser pulse wavelength, energy, width and focusing conditions via filamentation and laser-deposited energy density control [6,15,16] in order to reduce scattering losses and boost the related functional modalities (e.g., RID amplitude and thickness *T*). However, the current understanding of the role of filamentation and basic physical processes underlying ultrashort-pulse laser inscription of birefringent nanolattices in bulk dielectrics is still challenging and controversial [1,4,5,6,7,8,9,10,11,14]. As a result, this hinders current progress in developing laser inscription technology regarding RID homogeneity, scalability and robustness.

Furthermore, the essential birefringence characteristics of such nanolattices are usually incompletely characterized, indicating the only retardance magnitudes [1,3,14], while the corresponding RID values are rather roughly evaluated without [1] or with the very limited [3] thickness measurements, thus broadly ranging from 1 to 10^−3^ [1,3]. Such non-destructive thickness measurements are rather challenging for the embedded arrays of birefringent nanolattices, and cross-sectional scanning electron microscopy is usually performed just to visualize their orientation and periodicity in cross-cut samples [1,3,4,5,6,7,8,9,10,11] but not the overall thickness. As a result, the required informative simultaneous measurements of Ret and T magnitudes are very rare and are not representative [3]. Meanwhile, confocal photoluminescence and second-harmonic generation microscopy [14,17] were recently demonstrated to enable the non-destructive acquisition of the inscribed microstructure thicknesses, correlating with the corresponding cross-sectional atomic-force characterization analysis.

Moreover, the fundamental self-organization mechanism of such birefringent nanolattices is still questionable, undermining either ablative [11] or point-defect [14] material transport. In the latter case, just a few classes of materials—silicates, halogenides [18] and simple monoelemental substances (e.g., carbon in diamond [19])—support Frenkel “interstitial-vacancy” pair formation, involving oxygen, halogen and related atoms, respectively. Hence, the understanding of the chemical origin of the laser inscription process is the key issue in the harnessing of this technology for the other classes of transparent dielectric materials.

In this study, we explored the multi-parametric—laser wavelength-, pulsewidth- and energy-wise—ultrashort-pulse laser inscription of birefringent microstructures in fluorite in a pre-filamentation (linear/geometrical focusing) regime, revealing the important basic relationships between the retardance *Ret*, thickness *T*, RID magnitudes Δ*n* and laser parameters, as well as the important details of nanolattice self-organization in terms of material transport and its exposure-dependent dynamics. Our cross-sectional scanning electron microscopy analysis was performed to characterize the underlying nanoscale material transport processes. Far-field optical diffraction studies were conducted for the inscribing ultrashort laser pulses to analyze both the instantaneous plasma and accumulative structural patterns developing versus the laser exposure at diverse pulse energies.

## 2. Materials and Methods

In these experiments, we used fundamental- (1030 nm) and second-harmonic (515 nm) pulses of the Yb-fiber laser Satsuma (Amplitude Systemes, Pessac, France) with a repetition rate of 0–2 MHz and 4-μJ maximum pulse energy *E* in the TEM_00_ mode. Output full-width at half-maximum pulsewidth τ was varied for the corresponding fundamental 1030-nm radiation pulses using a grating compressor in the range τ = 0.3–3.8 ps and then frequency-doubled using a thin BBO-crystal plate, with the resulting pulsewidths measured by the autocorrelator AA-10DD-12PS (AVESTA Project, Russia). The 1030-nm and 515-nm laser pulses with variable energies up to 1 μJ (peak fluence < 10 J/cm^2^, pulsewidth-dependent peak intensity < 30 TW/cm^2^) at the 100-kHz repetition rate were focused in the workstation [20,21], with a 0.65-NA micro-objective into ≈2- and 1-μm wide spots (1/e-intensity diameter), respectively, at a depth of 80 μm below the top surface of the 2 mm thick fluorite (CaF_2_) slab with top/bottom optical windows (UV-mid-IR transmittance ≈90% in the spectral range of 0.15–9 μm) [14]. The fluorite samples were mounted on a PC-driven three-dimensional motorized translation stage (PRIOR, Cambridge, UK) and raster-scanned at the speed of 25 μm/s, providing either birefringent 3-line structures (length—200 μm) or 50-micron wide multi-line arrays with the scan direction along the laser polarization (see details elsewhere [14]). In the static mode of the sample, the diffraction patterns of the 515-nm, 0.3-ps laser pulses focused at different pulse energies in the range of 0.25–1.5 μJ with a 0.1-NA microscope objective and coming at the 25-Hz rate were acquired in the transmission far-field zone (distance from the focal point in the CaF_2_ slab ≈ 30 cm), using a color charge-coupled device (CCD) camera at the rate of 25 frames/s (see Section 3.3).

**Figure 1:**
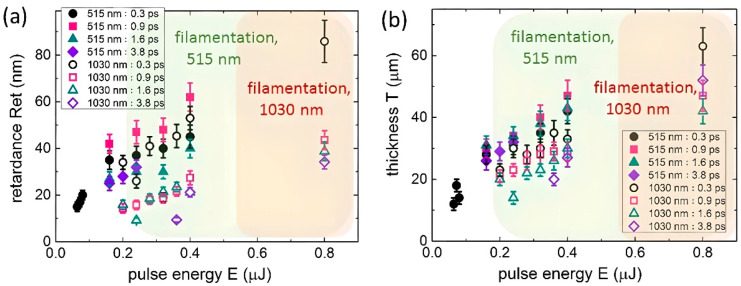
Retardance (**a**) and birefringent microstructure thickness (**b**) values versus pulse energy for different pulse widths (symbols of different shapes and colors) at 515 nm (solid symbols) and 1030 nm (open symbols) wavelengths. The green and reddish highlighted regions indicate the filamentation pulse energy ranges at these wavelengths, respectively, based on experimental results in [20].

In our characterization studies, birefringence in the modified region was analyzed by means of an Olympus BX-61 optical microscope (OLYMPUS, Tokyo, Japan) equipped with an Abrio IM 2.2 imaging system (Cambridge Research and Instrumentation, UK), with its operation principles described in [22]. The retardance value *Ret* (Figure 1a) was characterized by the spatial shift between orthogonally polarized extraordinary and ordinary waves that propagated through an anisotropic structure (fractional wavelength effect). It was expressed in units of wavelength as *Ret* = Δ*n* × *T*, where Δ*n* =|*n*_*e*_ − *n*_*o*_| for *n*_*e*_ and *n*_*o*_ being the extraordinary and ordinary refractive indexes, respectively, and *T* being the thickness of the birefringent structure along the laser-beam optical axis in the fluorite sample. The Abrio system operates at the laser-diode wavelength λ_probe_= 546 nm, acquiring the radial phase shift ≈*Ret*/λ_probe_. It also displays the orientation of the slow axis of the birefringent structure, i.e., the direction of the axis characterized by the highest value of the refractive index. The birefringent structures exhibited their slow optical axis aligned with the laser polarization, i.e., sub-wavelength periodical material modification occurs along the laser polarization similarly to LIPSS formation [10,11,14,23]. As a complementary characterization, cross-sectional green (540–580 nm) photoluminescence imaging at the 532-nm pump wavelength and magnification 100× was performed by means of a 3D-scanning confocal photoluminescence microscope Confotec 350 (SOL Instruments, Minsk, Belarus) to measure pulsewidth-dependent thickness *T* of the birefringent structures (Figure 1b). Moreover, the inscription pulse energies were compared to the threshold energies for the filamentation onset in this material at these wavelengths in [20] (shown in the figures by the highlighted regions).

For the sample preparation to reveal the topography and chemical modification of the birefringent tracks, their inscribed linear arrays were cut across the lines using a diamond blade disk Z09-SD3000-Y1-90 55 × 0.1 A2X40-L (DISCO, Tokyo, Japan) of an automated precision dicing saw DAD 3220 (DISCO, Tokyo, Japan). The cuts were ground with different Al_2_O_3_ powders, using grain sizes of 30, 9 and 3 μm, and then polished by ≈25 nm colloidal SiO_2_ nanoparticles using a polishing machine PM5 (Logitech, London, UK) until optical surface quality. Cross-sectional scanning electron microscopic (SEM) imaging of the separate microscopic birefringent tracks inside the bulk fluorite was performed using a low-vacuum electron microscope VEGA (SEM, TESCAN, Brno, Czech Republic), enabling the high-resolution visualization of the dielectric nanostructures without them changing. A chemical mapping and profiling microanalysis in the damage tracks was conducted using an energy dispersion X-ray spectroscopy module Xplorer (Oxford Instruments, Abingdon, UK) at different accelerating voltages (see Section 3.2).

## 3. Results and Discussion

### 3.1. Characterization of Birefringent Nanolattices

**Figure 2:**
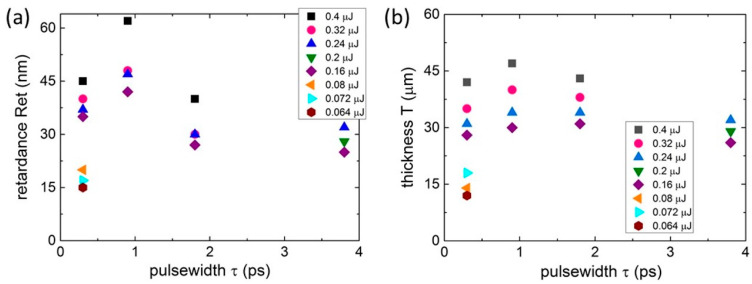
Retardance *Ret* (**a**) and thickness *T* (**b**) magnitudes versus pulsewidth τ at different 515 nm laser pulse energies (symbols of different shapes and colors). Maximal relative error bars ≤ 10%.

Our polarimetric characterization acquired the retardance magnitudes and monotonically scalable versus 515 nm laser pulse energy for all the laser pulsewidths both in the sub- and filamentation regimes (Figure 1a; the threshold energy for filamentation ≈ 0.2 μJ [20]). A similar *Ret* increase occurred in the case of the 1030 nm laser inscription, both in the sub- and filamentation regimes where the corresponding filamentation threshold energy was ≈0.5–0.6 μJ [20]. Inscription at the 515 nm wavelength exhibited considerably higher *Ret* magnitudes compared with the 1030 nm wavelength at the same pulse energies. Obviously, 0.9 ps laser pulses induced much higher *Ret* magnitudes at 515 nm, while 0.3-ps pulses produced a distinctly higher effect at 1030 nm. Interestingly, the energy dependences of the thickness *T* were similar at both of these wavelengths, with the minor (1030 nm) or even negligible (515 nm) difference as a function of the laser pulsewidth. These trends are presented below in Figure 2 for *Ret*(τ) and *T*(τ) curves, indicating the distinct correlation between the retardance magnitudes and thicknesses of the corresponding birefringent arrays of nanolattices in fluorite, i.e., the spatial scaling effect of the form-birefringence in this laser inscription mode.

Specifically, the *Ret*(τ) dependences at the 515 nm inscription wavelength for different pulse energies (see Figure 2a) showed a pronounced maximum of the magnitude at τ ≈ 0.9 ps. This was effective over the entire energy range and was more distinct at higher energies. This was consistent with the previous lattice-related observations of stronger etching in bulk-fused silica for the 1 ps laser pulse nanopatterning [24] and of ≈1 ps electron-ion thermalization in dielectrics [19,25]. The non-optical, lattice-related reason for this pulsewidth effect among the possible consequent optical, electronic, plasmonic, lattice and material transport phenomena could be illustrated by the corresponding weak, even and almost constant dependences *T*(τ) over the entire energy range (Figure 2b). Meanwhile, both the *Ret*(τ) and *T*(τ) curves exhibited the monotonous upraise versus pulse energy at the wavelength.

**Figure 3:**
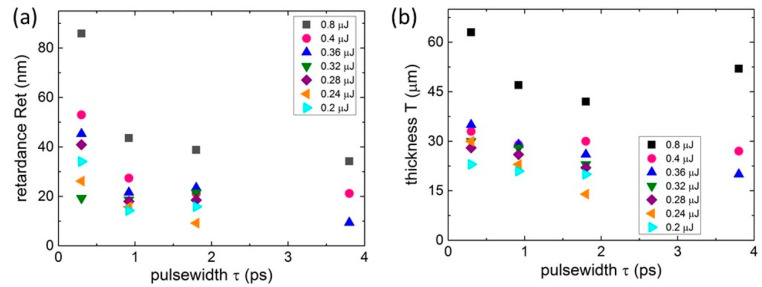
Retardance *Ret* (**a**) and thickness *T* (**b**) magnitudes versus pulsewidth τ at different 1030 nm laser pulse energies (symbols of different shapes and colors). Maximal relative error bars ≤ 10%.

In contrast, at the 1030 nm laser inscription wavelength the maximal Ret magnitudes at each pulse energy were achieved at the minimal pulsewidth and the maximal pulse energy (Figure 3a). In the same line, the corresponding *T*-magnitudes decreased versus τ at each pulse energy (Figure 3b) but were weaker than the corresponding *Ret* values. Simultaneously, *T*(τ) curves at the wavelength went almost together irrespective of the pulse energies until the maximal energy point of 0.8 μJ. This indicates that not the thickness, but the enhanced local energy deposition, i.e., the fluorite photoionization process, at the maximal pulse energy produced the maximal birefringence.

**Figure 4:**
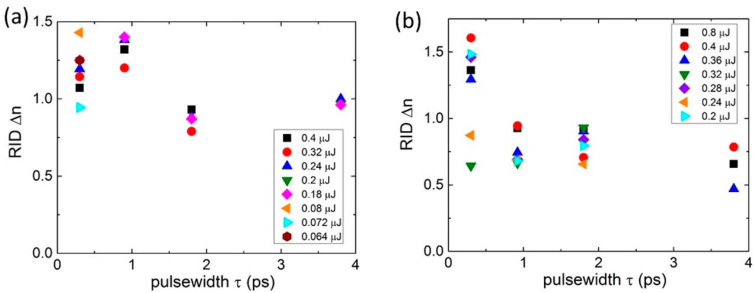
Refractive index difference (RID) Δ*n* = *Ret*/*T* versus pulsewidth τ at different 515 nm (**a**) and 1030 nm (**b**) laser pulse energies (symbols of different shapes and colors). Maximal relative error bars ≤ 10%.

Finally, the resulting refractive-index difference (RID) Δ*n* = *Ret*/*T* produced at the 515 nm (Figure 4a) and 1030 nm (Figure 4b) for the different pulse energies was analyzed as a function of the laser pulsewidth τ. First, one can see the maximal (by 10–20%), weakly energy-dependent RID values at the optimal 0.9 ps pulsewidth (see Figure 2). For the longer pulsewidths, the RID magnitudes tended to the lower constant, energy-independent level, providing robust pulsewidth- and energy-independent inscription of birefringent structures. Similarly, at the 1030 nm laser inscription wavelength, the produced RID values exhibited pulsewidth-sensitive but energy-independent trends with the maximum at the 0.3 ps pulsewidth and a 50% smaller level at the other pulsewidths and used energies (Figure 4b). Though these optimal pulsewidth-dependent inscription regimes are yet to be understood, the rather narrow RID variation range paves the way to the robust laser fabrication of predictable phase elements and devices, broadly scalable versus laser energy in terms of retardance.

### 3.2. Chemical Characterization of Inscribed Regions

**Figure 5:**
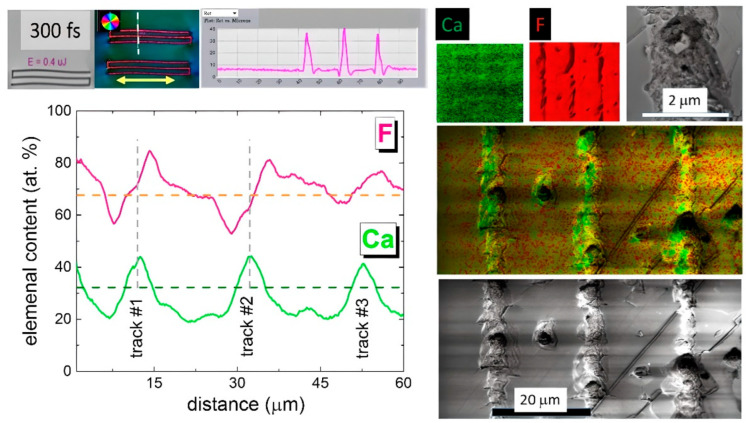
(Top line, left to right) Optical and polarimetric images of three-line buried microstructures inscribed by 0.3 ps, 515 nm laser pulses, and the corresponding retardance profile. Their cross-sectional EDX surface elemental color maps (Ca,F) and a magnified SEM image of one microstructure fragment. (Bottom line, right to left) Cross-sectional magnified and combined EDX elemental map of Ca (green color) and F (red color) across the three-line structure and its SEM image. Profiles of Ca and F across the three-line structure.

The intriguing issue of ultrashort-pulse laser inscription is the driving force for nanoscale material transport in the separate birefringent nanolattices and their bulk arrays. This could be either laser ablation with the formation of nano- [17] and microcavities [13] or the drift of charged and neutral point defects in transient electron-hole plasma-induced electrical fields of dynamically curved energy bands [26] and fields of excited hot acoustic phonons [19,27] (local mechanical stresses). In the first case, in fluorite—both calcium Ca and fluorine F—chemical components should be depleted in the ablation zone [28]. In the second case, different distributions of these components could occur depending on the signs of the charge states of defects and the signs of the specific strain per defect (e.g., positive for interstitials and negative for vacancies). In this study, cross-sectional SEM visualization and EDX elemental mapping were performed on the saw-cut and polished birefringent 3-line structures in order to envision the underlying material transport processes (Figure 5).

Specifically, in the elemental EDX maps of Ca and F in Figure 5, one can see the depletion of fluorine and the enrichment by Ca in the microstructure cross-sections, while the combined map indicates some redistribution of fluorine around the lines. More exactly, cross-profiling of the map indicates that the Ca distribution is modulated by ≈10 at.% around the stoichiometric value of 33 at.%, indicating the in-flow of Ca atoms into the laser tracks at the expense of its content in the surrounding material. This effect is known for ultrashort-pulse laser inscription of densified spots in silica glasses [29] but was never mentioned for fluorite. Furthermore, the corresponding F-content profiles demonstrate the alternate variation within the tracks, with the asymmetric increase on one side and a decrease on the other side in the radially-symmetrical inscription geometry; this effect is unknown and should be understood yet. One can see partial, non-ablative depletion (F) or enrichment (Ca,F) within the laser-inscribed microstructures, with clear evidence of the different transport for the various chemical components.

### 3.3. Far-Field Optical Diffraction In Situ Characterization of Instantaneous Plasma and Accumulative Structural Changes

**Figure 6:**
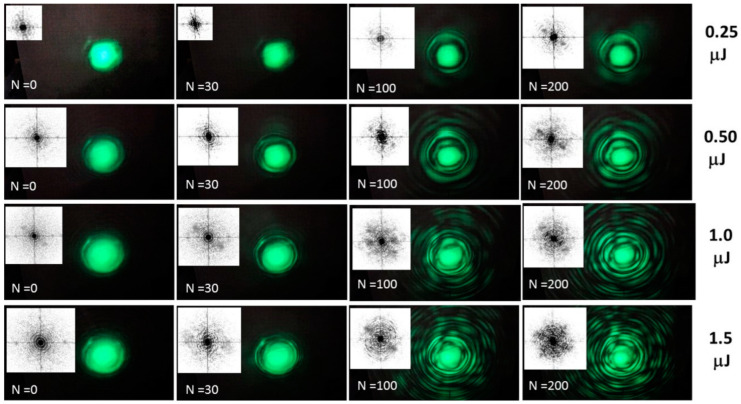
Far-field diffraction patterns of 515 nm, 0.3-ps laser pulses at different energies of 0.25–1.5 μJ (filamentation threshold energy—0.2 μJ) and exposures N = 0–200 (N = 0 corresponds to the first incident laser pulse), and their black/white spatial 2D FFT spectra. The frame size is 17 × 10 cm.

Far-field optical-diffraction probing of the prompt plasma and accumulated material microstructures, using the inscribing 515 nm, 0.3 ps laser pulses, was performed in situ in the static exposure mode both near the filamentation threshold (0.2 μJ) and in the filamentation regime well above this threshold at the pulse energies of 0.5, 1.0 and 1.5 μJ. Simultaneously, laser exposure was varied in all these regimes to reveal the accumulative structural changes inside fluorite independently of the filamentation [30]. Meanwhile, laser exposure-dependent atomistic damage could decrease the filamentation threshold energy, as demonstrated in [19]. The corresponding diffraction images and their 2D Fast Fourier-Transform (FFT) spectra are presented in Figure 6, characterizing both the filamentary conical emission (radial ring-like patterns both in the images and spectra) and diagonal FFT peaks at the higher N, implying some periodicity in diffraction patterns and the underlying focal or filamentary material structures. The spatial scales of the plasma and material structures were evaluated as Δ ~ λ_ins_ × (*L*/2*r*), where the inscribing central laser wavelength λ_ins_ ≈ 515 nm, diffraction length to the CCD camera *L* ≈ 30 cm and *r* is the radius of the pattern.

Specifically, in Figure 6, at the minimal near-threshold pulse energy of 0.25 μJ, one can see the exposure-dependent transformation of the initial diverging Gaussian beam into the circular patterns (N = 100) and separate peaks (N = 200). The N-dependent emerging circular patterns could indicate the microscale damage cavity, while the spectral peaks could characterize micron-scale radial periodic structures in the focal region or the emerging filament. In contrast, at higher above-threshold pulse energies, representing the filamentation regime (see the (multi)ring FFT patterns, starting at N = 0 in Figure 6), more complex multi-ring patterns appear in the diffraction images, giving rise to the more sophisticated—multi-ring or multi-peak—spectral patterns, which are yet to be understood in terms of laser pulse self-action during the inscription process [30,31]. Their analysis is also ongoing in the optical spectral (wavelength) domain in order to resolve non-linear optical, plasma and material effects.

Finally, the ultrashort-pulse laser inscription process under study in fluorite appeared as a very sophisticated physical phenomenon involving non-linear and non-equilibrium optical, plasma and material effects, as well as their cross-linking effects (plasma absorption [32], plasma-mediated defect generation [18], etc.). Meanwhile, stronger research efforts will facilitate the harnessing of this versatile technology both in additive [33] and subtractive [17,24] modes.

## 4. Conclusions

In this study, the important relationships between the birefringence and the thickness of laser-inscribed arrays of birefringent nanolattices in bulk fluorite and key inscription parameters—laser wavelengths (515 and 1030 nm), (sub)μJ pulse energies (both sub- and filamentation regimes) and (sub)picosecond pulsewidths (0.3–3.8 ps)—were established for the first time. Broad scalability of retardance magnitudes versus laser pulse energy was revealed at both these laser wavelengths, exhibiting the different optimal 515 nm and 1030 nm pulsewidths of 0.9 ps and 0.3 ps, respectively. The accurately measured weakly wavelength- and pulsewidth-dependent refractive-index changes ~10^−3^ in bulk nanolattices demonstrate the robustness and scalability of ultrashort-pulse laser inscription, indicating its non-ablative and accumulative origin, supported by electron-microscopy chemical microanalysis and dynamic in situ optical-diffraction studies. These observations enable facile downscaling/upscaling of retardance magnitude in the laser-inscribed embedded phase waveplates and the more sophisticated integrated bulk micro-optical interferential-polarizing devices, providing the essential background for advanced modeling and the envisioning of the underlying physical laser inscription mechanisms in fluorite and, potentially, other dielectrics.

## Data Availability

The data supporting the reported results are presented in part in Supplementary Data and can be also obtained from the authors.

## Supplementary Materials

The following supporting information can be downloaded at: https://www.mdpi.com/article/10.3390/nano13061133/s1.
