# Supplementary material for: Multi-Parametric Birefringence Control in Ultrashort-Pulse Laser-Inscribed Nanolattices in Fluorite
Source: Nanomaterials (Basel). 2023 Mar 22;13(6):1133. doi: 10.3390/nano13061133 (PMC10058163; doi:10.3390/nano13061133)
Supplement: Supplementary file 1 [file nanomaterials-13-01133-s001.zip › nanomaterials-2270907-supplementary.pdf]

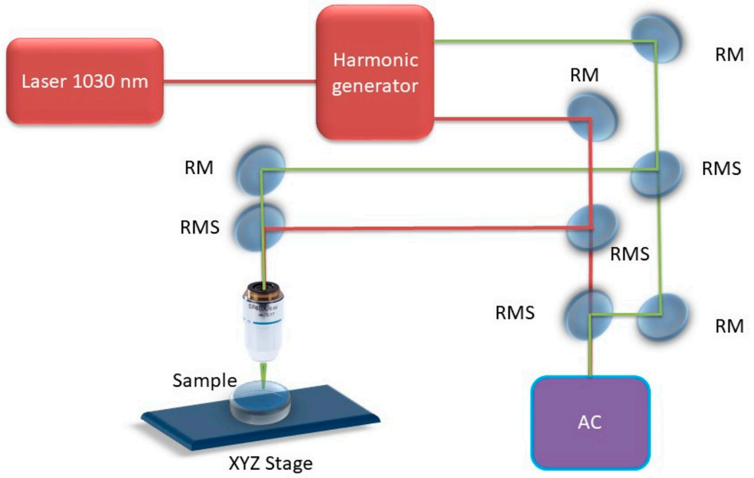

Figure S1. Schematic of the laser setup: 1) Satsuma laser system; 2) harmonic generator (second harmonic generation at 515 nm); 3) RM – highly-reflective mirror; 4) RMS – folding highly-reflective mirror; 5) AC – autocorrelator; 5) XYZ stage; 6) Sample – slab of calcium fluoride (monocrystal).

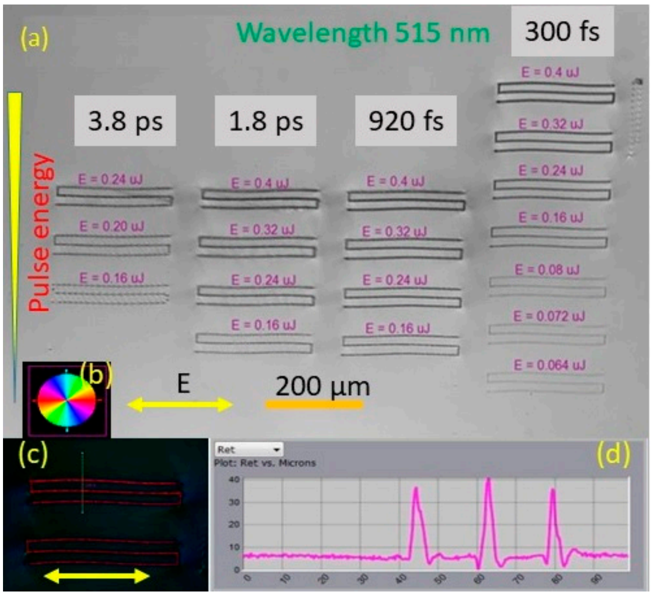

Figure S2. a) Optical image of 3-line structures inscribed (at a wavelength of 515 nm) in fluorite at the 50-micron depth at different pulsewidths and energies (the yellow arrow indicates the laser polarization direction.). (b) Color map (color describes the direction of the slow axis) (c) Typical optical and polarimetric (Abrio) pseudocolor images of the structures inscribed at 0.3-ps pulsewidth and energies of 0.4 (top) and 0.32 (bottom) μJ (the red color of the lines shows their horizontal slow axis direction according to the map). (d) Retardance variation across the three-line structure in (c, top).

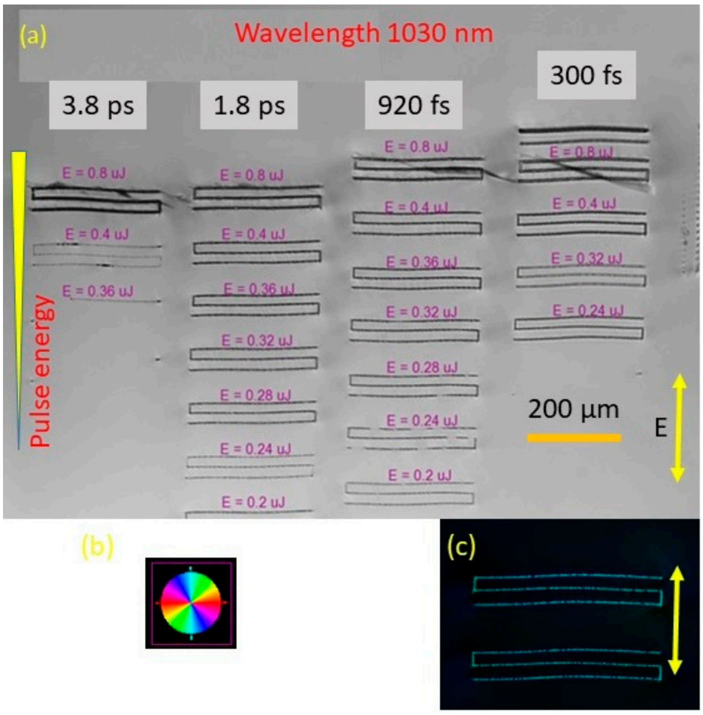

Figure S3. a) Optical image of 3-line structures inscribed (at a wavelength of 1030 nm) in fluorite at the 50-micron depth at different pulsewidths and energies (the yellow arrow indicates the laser polarization direction.). (b) Color map (color describes the direction of the slow axis) (c) Typical optical and polarimetric (Abrio) pseudocolor images of the structures inscribed at 0.3-ps pulsewidth and energies of 0.36 (top) and 0.28 (bottom) μJ (the blue color of the lines shows their horizontal slow axis direction according to the map).
